# Migraine: a major debilitating chronic non-communicable disease in Brazil, evidence from two national surveys

**DOI:** 10.1186/s10194-019-1036-6

**Published:** 2019-08-01

**Authors:** Mario Fernando Prieto Peres, Luiz Paulo Queiroz, Pedro Sampaio Rocha-Filho, Elder Machado Sarmento, Zaza Katsarava, Timothy J. Steiner

**Affiliations:** 1 Hospital Israelita Albert Einstein, Rua Joaquim Eugenio de Lima, 881 cj 708, São Paulo, SP Brazil; 2 Instituto de Psiquiatria, Hospital das Clínicas da Faculdade de Medicina da USP, Rua Joaquim Eugenio de Lima, 881 cj 708, Sao Paulo, SP Brazil; 3 Universidade Federal de Santa Catarina, Florianópolis, Santa Catarina Brazil; 4 Department of Neuropsychiatry, Universidade Federal de Pernambuco, Recife, Pernambuco Brazil; 5 Universidade Federal Fluminense, Rio de Janeiro, Brazil; 6 Evangelical Hospital Unna, Unna, Germany; 7 Departmentof Neurology, University of Duisburg-Essen, Essen, Germany; 8 EVEX Medical Corporation, Tbilisi, Georgia; 9 IM Sechenov First Moscow State Medical University (Sechenov University), Moscow, Russian Federation; 10 Department of Neuromedicine and Movement Science, NTNU Norwegian University of Science and Technology, Trondheim, Norway; 11 Division of Brain Sciences, Imperial College London, London, UK

**Keywords:** Migraine, Non-communicable diseases, Public health, Health surveys, Epidemiology, Health care, Brazil, Global campaign against headache

## Abstract

**Background:**

Even though migraine and other primary headache disorders are common and debilitating, major health surveys in Brazil have not included them. We repair this omission by combining data on non-communicable diseases (NCDs) in the Brazilian National Health Survey (PNS) 2013 with epidemiological data on migraine prevalence and severity in Brazil. The purpose is to rank migraine and its impact on public healthh among NCDs in order to support public-health policy toward better care for migraine in Brazil.

**Methods:**

Data from PNS, a cross-sectional population-based study, were merged with estimates made by the Brazilian Headache Epidemiology Study (BHES) of migraine prevalence (numbers of people affected and of candidates for migraine preventative therapy) and migraine-attributed disability.

**Results:**

Migraine ranked second in prevalence among the NCDs, and as the highest cause of disability among adults in Brazil. Probable migraine accounted for substantial additional disability. An estimated total of 5.5 million people in Brazil (or 9.5 million with probable migraine included) were in need of preventative therapy.

**Conclusion:**

On this evidence, migraine should be included in the next health surveys in Brazil. Public-health policy should recognize the burden of migraine expressed in public ill health, and promote health services offering better diagnosis and treatment.

## Introduction

Chronic non-communicable diseases (NCDs) are a principal concern in public health. This is as true in Brazil [1] as elsewhere [2]. Among NCDs, neuropsychiatric (neurological and mental health) disorders have been identified as the single largest group of contributors to public ill health [2, 3], while pain conditions also play a important role [4]. Their impact is not limited to deaths attributable to NCDs (72% in Brazil [5], but is manifested also in high levels of disability, in Brazil [1, 5] and globally [2, 3]. The economic burdens on individuals and society are expressed in heavy direct and indirect costs [1, 6].

Brazil has implemented public-health policies to reduce the burden of NCDs, but the targets are restricted to hypertension, diabetes, cardiovascular diseases (including stroke) and mental health disorders. The last do not include neurological conditions, which remain low-priority. These policies are driven by NCD surveillance: the National Health Survey (PNS-Pesquisa Nacional de Saúde) in 2013 gathered information on distribution and magnitude of these selected NCDs, and identified risk factors and the social, economic and environmental associations [7]. The purpose was to support better preventative measures [7].

Primary headache disorders, in particular migraine, are common and debilitating conditions [8]. Migraine is now recognized as the second highest cause of disability worldwide, first in those under 50 years of age [9]. In Brazil, migraine has been found in 15.2% of the population [10], tension-type headache in 13% [11] and headache disorders characterized by headache occurring on 15 or more days per month in 6.9% [12].

Nevertheless, PNS, the most comprehensive survey on health and its determinants ever held in the country, did not encompass headache disorders [7], and public-health policies based on PNS do not target them. Headache management in the population is suboptimal, with limited access to preventative treatments: 7.7% of candidates for prophylaxis (MIDAS score > 10) receive some, but only 2.6% adequately according to prophylaxis guidelines [13, 14]. Yet a recent study showed promising results with non-pharmachological interventions in a low-income, underserved community in Brazil [15].

This study endeavours to rectify the omission from PNS, by combining PNS data on NCDs [7] with epidemiological data on migraine prevalence and severity in Brazil [10–12]. The purpose was to rank migraine and its attributed disability among NCDs, so supporting public-health policy toward better care for migraine in Brazil.

## Methods

### National Health Survey (Pesquisa Nacional de Saúde - PNS)

PNS was a household survey developed in partnership between the Health Surveillance Secretariat (SVS/MS) of the Ministry of Health, Oswaldo Cruz Foundation (Fiocruz) and the Brazilian Institute of Geography and Statistics (Instituto Brasiliero de Geografia e Estatistica: IBGE). Conducted between 2013 and 2014, it is the most complete survey on health in Brazil. It adopted simple randomized cluster sampling in three stages: census tracts, households and residents aged ≥18 years, including one individual per household. Interviews were completed by trained professionals with the aid of handheld computers. Full details of the sampling design and methods have been described elsewhere [7, 16, 17]. The questionnaire and data are available at: http://www.pns.icict.fiocruz.br/arquivos/Novos/Questionario%20PNS.pdf.

PNS was approved by the National Research Ethics Committee, under protocol number 328.159, on 26 June, 2013. All participants were informed and gave signed consent.

### NCD indicators

Participants in PNS were asked about past physician-diagnoses with regard to a range of NCDs, about self-reported spine or lung disorders, and about mental-health professional diagnoses of depression and other mental-health disorders (Table 1). Previous publications showed the validity of a self-reported history of doctor diagnosed NCD in other surveys [18, 19] and in PNS [20, 21]. Hypertension may be overestimated in self-reports by 10% [20].

**Table 1:** Enquiry into non-communicable diseases in the National Health Survey (PNS)

Disease	Enquiry	Response options
Hypertension	“Has any doctor given you the diagnosis of hypertension (high blood pressure)?”	yes; yes, but only during pregnancy (for women); no
Diabetes; Heart disease; Lung disease; Stroke; Asthma; Arthritis or rheumatism; Work-related musculoskeletal disorders (WMSDs); Cancer; Chronic kidney disease (CKD)	“Has any doctor given you the diagnosis of ...?”	yes no
Spine disorders	“Do you have any chronic back problem, such as back pain, neck pain, low back pain or sciatica, vertebrae or disc problems?”	yes no
Depression	“Has a doctor or mental health professional, such as a psychiatrist or psychologist, ever given you the diagnosis of depression?”	yes no
Other mental illnesses: schizophrenia; bipolar disorder; psychosis; obsessive-compulsive disorder (OCD)	“Has a doctor or mental health professional, such as a psychiatrist or psychologist, ever given you the diagnosis of ...?”	yes no

PNS captured proportions (%) and total numbers of participants, 18 years or older, who reported positively to each. Proportions reporting high or very high degrees of limitation in activities of daily living due to each of these conditions were also captured.

### Brazilian headache epidemiological study (BHES)

This was a population-based cross-sectional study interviewing randomly selected individuals by telephone. Sample size was calculated expecting a migraine prevalence of 20%, with 2% error and 95% confidence. The estimated number of 1,537 was inflated by 2.5 design effect, to yield *N* = 3,843 subjects to be interviewed [10–12]. Trained lay interviewers followed a structured questionnaire validated for the diagnosis of primary headache disorders according to the International Classification of Headache Disorders (ICHD-II) [22]. The headache questionnaire has been validated previously [23], additional validation was performed in 50 individuals in BHES showing that lay interviews were satisfactory [11].

Migraine was diagnosed when all diagnostic criteria were met, and probable migraine when all criteria were met but one. Both were included in “all migraine”.

One-year prevalence of each was estimated. Impact on health was established using MIDAS (Migraine Disability Assessment Scale), which estimates lost productive time [24]. For each diagnosis, we applied two thresholds of impact. First, those with MIDAS scores ≥10 were expected to be candidates for migraine prevention (having at least 3 attacks per month), and considered disabled. Second, those with MIDAS grade IV, the highest grade possible, were counted as severely affected.

This study was approved by the ethics committee of Albert Einstein Hospital, São Paulo, Brazil.

## Results

PNS interviewed 60,202 individuals older than 18 years from 64,348 households, a participating proportion of 91.9%. The total adult Brazilian population from which this sample was drawn was 146,176,000 in 2013, but we based estimates of migraine and other diseases on the current (2018) general population of 207 million, or 150,539,000 adults (https://www.ibge.gov.br/apps/populacao/projecao/). BHES completed interviews of 3,848 participants, aged 18–79 years, from all 27 States and all five geographical regions of Brazil.

Participants in PNS reporting at least one NCD were 45.1% (50.4% in females, 39.2% in males). Table 2 shows the proportions of individuals who reported each NCD, sub-proportions with high disability, and derived population prevalences of those disabled with each NCD, according to PNS. It also shows prevalences of migraine, probable migraine and all migraine according to BHES.

**Table 2:** Proportions (%) reporting chronic non-communicable diseases, sub-proportions (%) reporting high disability, and derived population prevalences of disease with high disability (%) [data from Brazilian National Health Survey, 2013]; prevalences of migraine and probable migraine, and those severely affected [data from Brazilian Headache Epidemiology Study], with estimates for the total Brazilian population

Disease	Proportion reporting (%)	Sub-proportion (%) with high disability		Population prevalence of disabled (%)	Estimated numbers disabled* (N)
Hypertension	21.4	4.7		1.01	1,472,250
Diabetes	6.2	7.0		0.43	635,272
Asthma	4.4	15.7		0.69	1,011,165
Depression	7.6	11.8		0.90	1,312,700
Other mental health disorder	0.9	37.6		0.34	495,336
Heart disease	4.2	13.5		0.57	829,952
Stroke	1.5	25.5		0.38	559,888
Arthritis	6.4	17.1		1.09	1,601,939
Any spine disorder	18.5	16.4		3.03	4,441,048
WMSD	2.4	15.7		0.38	551,545
Cancer	1.8	10.3		0.19	271,381
Chronic kidney disease	1.4	11.9		0.17	243,862
Pulmonary disease	1.8	10.0		0.18	263,477
Migraine	15.2	MIDAS ≥10	24.7	3.75	5,495,541
		MIDAS IV	12.3	1.87	2,736,646
Probable migraine	14.3	MIDAS ≥10	19.3	2.76	4,039,831
		MIDAS IV	7.1	1.01	1,477,783
All migraine	29.5	MIDAS ≥10	22.0	6.51	9,535,372
		MIDAS IV	9.6	2.88	4,214,429

*WMSD* work-related musculoskeletal disorders, *MIDAS* Migraine Disability Assessment Scale; *estimates based on Brazilian population of 207 million (https://www.ibge.gov.br/apps/populacao/projecao/)

Hypertension was highest in prevalence (21.4%) among the NCDs, followed by spine disorders (18.5%), which were well clear of all others (Table 2). However, all migraine (29.5%) outdistanced hypertension by a large margin. Other mental health disorders were, relatively, the most disabling of the NCDs, with 37.6% of those affected reporting high or very high disability, followed by stroke (25.5%). All other NCDs came well below these. Migraine (24.7%) was just below stroke. In terms of population prevalence and absolute number with high or very high disability, spine disorders (meaning back pain, neck pain, low back pain or sciatica, vertebrae or disc problems) far exceeded all other NCDs: prevalence 3.03%, 4.4 million affected. Hypertension was next, with only one third of these levels (Table 2). Migraine, however, clearly exceeded spine disorders: 3.75%, and 5.5 million disabled. All migraine almost doubled these: 6.51%, 9.5 million. Those severely disabled (MIDAS grade IV) (2.88%; 4.2 million) were, on their own, just below spine disorders.

Assuming migraine had been included as a NCD in PNS, and the same prevalences found as in BHES, it would have ranked as the most common NCD. Assuming MIDAS grade IV described impact equivalent to disability rated by participants in PNS as high or very high, migraine would have ranked second in Brazil. If MIDAS ≥10 was indicative of high disability, migraine would have ranked first. Including probable migraine (all migraine) would have substantially inflated the migraine estimates but not altered the rankings.

An estimated 5.5 million people in Brazil (or 9.5 million if probable migraine is included, as it should be [25]) are apparently in need of migraine preventative therapy.

## Discussion

Migraine is among the most prevalent NCDs in Brazil, and the most disabling in terms of numbers reporting impact equated with severe disability. Probable migraine is a substantial contributor to the disability burden. Probable migraine has often been neglected in epidemiological studies but, at least in those concerned with public health, it should not be [25]. It is more probably migraine than any other disorder, and BHES showed its impact is not much below that of migraine meeting all ICHD criteria [10]. The burden of migraine is greatly underestimated if probable migraine is ignored [25] .

Although the methodology used here was imperfect, combining data from two very different surveys and extrapolating numbers, the findings mirror those obtained in the Global Burden of Disease (GBD) studies: migraine is among the most prevalent disorders worldwide, and the second most disabling behind low back pain [2, 9]. Migraine affected 1·04 billion people worldwide causing 45·1 million years lived with disability (YLD) in 2016 [26], the average global prevalence estimated to be around 12% [27]. In Brazil, migraine prevalence was 15.2%, tension-type headache 13%, and daily headaches in 6.9%. Probable migraine affected another 14.3%, combined with migraine, 29.5%, both higher than most epidemiological studies worldwide, in the US (12.8% migraine, 4.8% probable migraine) [28] and Europe (11.2% and 10.1%) [29]. In Brazil, other studies showed high numbers of probable migraine prevalence in different populations [30–32], therefore, a high global burden of migraine is trully substantial in Brazil.

The need for prevention is another key topic for health care policy planning in headache disorders [33]. We used MIDAS > = 10 as a cutt of for at least a > = three headache days monthly frequency, indicative in most guidelines worldwide of starting prophylaxis [13, 34]. In Brazil, 3.5% of migraine patients presented with a MIDAS > = 10, similar to 3.0% found in the US population [33]. In the US, however, only 13% were taking daily treatment for headaches compared to 2.6% in Brazil [14].

Quite clearly, migraine and other headache disorders should be prioritized in health-care policies. This message has already been clearly sounded by the World Health Organization [35], and reiterated multiple times by the Global Campaign against Headache [9], and here we present striking evidence from Brazil to support it. For better estimates to inform policy, migraine should be included in the next health surveys in Brazil. Spine disorders should probably be studied in more detail, since there are probable overlaps between pain disorders, not to mention comorbidities with sleep and mental health disorders.

One study limitation has been mentioned: we merged two studies with different methodologies. The 5 years between their data collections are unlikely to have had much influence. In PNS, there were considerable diagnostic uncertainties, likely to have had some impact on prevalence estimates (in either direction). BHES, on the other hand, used a validated diagnostic instrument. In its favour, PNS was a door-to-door survey whereas BHES relied on telephone interviews. We made assumptions about the relationship between disability and MIDAS scores, but this was, probably, more reliable than the self-reporting of high or very high degrees of limitation in activities of daily living used in PNS. Since migraine is under-diagnosed, PNS numbers regarding migraine are probably under-estimated.

## Conclusion

Migraine is the second most common NCD in Brazil, and the most disabling NCD. It is illogical that health policy ignores it. Migraine should be part of the next health surveys in Brazil, to inform public-health strategies promoting better diagnosis and treatment.

## Data Availability

The data used for the analysis will bem ade available upon request

## Abbreviations

BHES: Brazilian Headache Epidemiological Study
GBD: Global Burden of Disease
ICHD: International Classification of Headache Disorders
MIDAS: Migraine Disability Assessment Scale
NCD: Non-communicable diseases
PNS: National Health Survey (Pesquisa Nacional de Saúde)
